# “We Represent a Definite Social Class”: The Class Identities and Resources of American Religious Groups in the Roaring Twenties

**DOI:** 10.1111/1468-4446.70063

**Published:** 2026-01-31

**Authors:** Tessa Huttenlocher, Melissa Wilde

**Affiliations:** ^1^ University of Chicago Chicago Illinois USA; ^2^ University of Pennsylvania Philadelphia Pennsylvania USA

**Keywords:** class identity, comparative historical, mixed methods, organizational identity, religion, social class

## Abstract

Class identity is a crucial sociological concept, but is only ever measured at the individual level. In this paper, we ask: do groups have class identities? And do those class identities correspond with material resources? To answer these questions, we examine data from 31 of the most prominent American religious denominations in the early 20th century. We find that religious groups expressed palpable class identities in their denominational periodicals, and that these identities were broadly aligned with these groups' material resources. This study suggests that studying class identity at the group level can deepen our understanding of inequality—both in the highly stratified field of organized religion, and among organizations and other social groupings more generally.

## 1 Introduction

Scholars have long recognized that class identity, defined as the subjective experience of one's objective social class position, plays a crucial role in politics and social movements. However, despite the key role of groups in these processes, class identity is rarely theorized at the level of groups. In this paper we ask: can groups have class identities? And do those identities correspond with material resources? Using data from America's largest and most prominent religious denominations in the early 20th century, we find that American religious groups had distinct and palpable class identities which reflected their structural positions, with religious periodicals regularly formulating generalizations about the “kinds of people” their denomination served.

Our analysis of American religious periodicals revealed three kinds of class identities. The *Professionals* tended to call themselves by this moniker, and identified themselves as privileged, both explicitly as well as through their discussions of topics like organized philanthropy, higher education, and labor conflicts. Professionals also all turned out to be urbanites—something which emerged from our analysis, and which we discuss below. In contrast, the *Property Holders* tended to publish articles identifying themselves as landowners or agricultural businessmen, discussing issues of rural life and work from the perspective of farm and mine owners. The Property Holders also wrote about higher education with familiarity and espoused philanthropy, but in distinctive ways that reflected their more rural character. Finally, the *Laborers* discussed the challenges that their poor and working‐class members faced, whether in urban or rural settings. In discussions of poverty and labor conflict, these denominations sympathized with workers, and either didn't mention topics like higher education or philanthropy, or did so critically. We argue that our measures of group‐level class identity are useful for researchers seeking to understand the role of class identity in the American religious field, and in organizational fields more broadly.

### 1.1 Class Identity and Material Resources

Class identity can be understood as the subjective experience of one's objective class position. Marxist scholar Erik Olin Wright explained that,

To identify with a particular class is to perceive the world in certain categories, probably to hold some theories about the causes and consequences of class membership, and to hold at least some evaluative sense of interests tied to that class.

(Wright 1985, 254)

Wright argues that identification with a class is experienced subjectively, but patterned according to objective material class positions, with individuals having different varieties of class consciousness depending on their ownership of the means of production and their control over the use of organizational assets.

Since Marx and Engels' conceptualization of “false consciousness” (Eyerman 1981), researchers have studied the relationship between material resources and class identity, finding that while people can sort others into social classes accurately, their self‐categorizations are less precise (Jackman and Jackman 1973, 1985; Hout 2008), with nearly, two‐thirds of the American upper middle class having “deflated” perceptions of their own social class, and one‐third of the American working class having “inflated” perceptions (Sosnaud et al. 2013).

Assessments of one's social class can be swayed by political affiliation (Egan 2020), urban v. rural location (Pauli 2020), and the social class of one's friends (Hodge and Treiman 1968), coworkers (Lockwood 1989) and spouse (Abbott 1987; Davis and Robinson 1988; Baxter 1994). Schwadel (2024) finds that religious affiliations can also distort class identification, with Conservative Christians being more likely to identify as working class, regardless of education or income. Individuals' class identities are therefore imperfectly representative of their material resources and objective class positions.

Class identity (mis)apprehension is often used to explain political mobilizations and social movements (Weber [1910] 1978; Eidlin and Kerrissey 2018), phenomena that have significant group‐level components (Armstrong and Bartley 2017). However, class identity remains understudied as a characteristic of groups and organizations. Even the “collective identity” concept used by social movement theorists refers to “an *individual's* cognitive, moral, and emotional connection to a broader community, category, practice, or institution” (Polletta and Jasper 2001, 285, *emphasis added*). By systematically investigating the class identities and material resources of denominations in the American religious field, we demonstrate that the concept of class identity is applicable to groups, not just individuals.

### 1.2 Class Identity at the Group‐Level

Organization theorists have explored the topic of group‐level identity through the concept of “organizational identity,” which Albert and Whetten (1985) define as that which members broadly recognize as being “central, distinctive, and enduring” about an organization (266). Much like individual‐level identities, organizational identities affect organizations' decision‐making (Dutton and Dukerich 1991) and can change over time (Gioia et al. 2000; Glynn 2000; Elsbach and Kramer 1996). However, since these works have largely focused on organizations' efforts to affirm positive identities or repair spoiled ones (McDonnell and King 2013), they have mostly examined positive, legitimizing elements of organizational identity, not the many more complex, ambivalent, stigmatized, or downplayed identities an organization may have.

Nevertheless, there is growing consensus that organizational identities are connected to the reproduction of social inequalities (Tomaskovic‐Devey and Avent‐Holt 2019). Like individuals, organizations are racialized (Poon et al. 2024; Diefendorf and Pascoe 2023; Ray 2019; Wooten and Couloute 2017), gendered (Ruspini 2021; Boogaard and Roggeband 2010; Acker 1990, 2006), ethnicized (Boogaard and Roggeband 2010), “sexualed” (Hearn 2015)…and, yes, classed (Gray and Kish‐Gephart 2013; Côté 2011).

However, these studies mostly focus on the marginalization of individuals whose *personal* identities do not align with their organizations'. Ultimately, this means that the existing literature has neglected the “organization” part of organizational identity, obscuring the ways that organizations function as social actors in and of themselves (King et al. 2010).^1^ Also, as Desmond and Clevenstine (2025) have argued, a focus on individual‐level inequalities can obscure the role of powerful institutions and organizations in perpetuating inequality. Finally, as Foreman and Whetten (2016) have noted, studies of organizational identity overwhelmingly tend to have an N of 1, precluding a broader understanding of organizational identity variation within organizational fields. In this paper, we correct these oversights by examining the class identities of 31 prominent religious organizations in the highly stratified American religious field of the early 20th century.

### 1.3 Social Class in American Religious Groups

Early sociologists were much more attuned to class differences among American religious groups than we are today (Weber [1905] 2001; DuBois [1899] 1996; Niebuhr 1929; Drake and Cayton 1993; Demerath 1965). For example, Hadley Cantril (1943) found that “Catholics are poorer and less well‐educated than Protestants” (575), and Liston Pope (1948) noted that the Baptists were the “least educated” of American Protestants, and Congregationalists the most (8). Mid‐century community studies regularly pointed out the ways that people of different social classes gravitated toward different denominations (Varenne 1977; Pope 1976; Lynd and Lynd 1982; Vidich and Bensman [1958] 1968; Lenski 1961; Baltzell [1958] 1992). However, attention to religious stratification faded in the latter half of the 20th century, as debates over secularization came to dominate the sociology of religion and many prominent scholars predicted the decline of religious beliefs and institutions (Berger 1967; Bruce 1990; Wilson 2016).

More recently, however, a growing field of scholarship has returned to the idea of religion as a site for social class formation and reproduction (McCloud 2007; McCloud and Mirola 2008; Keister and Sherkat 2014; Schwadel 2016). Davidson and Pyle (2011) found that the American upper‐class is still dominated by what E. Digby Baltzell dubbed the “Protestant Establishment” (Baltzell 1964). Other patterns of religious inequality remain robust (Darnell and Sherkat 1997; Park and Reimer 2002; Smith and Faris 2005; Boddie et al. 2012; Keister and Sherkat 2014; Pyle and Davidson 2014; Wilde et al. 2018), with evidence that religion affects individuals' social class outcomes indirectly through early life orientations toward education (Horwitz 2022; Lehrer 2010; Sikkink and Hill 2016), women's labor market participation (Lehrer 2004; Glass and Jacobs 2005), timing of marriage and childbearing (Fitzgerald and Glass 2008), and wealth accumulation and transmission (Keister 2003, 2008, 2011). Additionally, religion has been and continues to be highly segregated by race (DuBois [1899) 1996; Lincoln and Mamiya 1990; Pattillo‐McCoy 1998; Emerson and Smith 2001; Wood 2002; McRoberts 2003), geography (Hill 1985; Stump 1998; Koçak and Carroll 2008; Silk and Walsh 2008) and immigration (Herberg 1955; Niebuhr 1929; Cadge and Ecklund 2007), all of which are entangled with social class.

Religious groups act as sites where all of these intersecting inequalities play out—an insight from a growing body of work called “complex religion” (Wilde 2017; Wilde and Tevington 2017) which posits that inequality is inextricably intertwined with religion in ways that explain phenomena often ascribed to theology (Wilde et al. 2018). While intersections between the social class of American religious groups and other axes of inequality have been discussed elsewhere (Wilde et al. 2025) we still know little about how these material realities played out in groups' subjective identities.

## 2 Data and Methods

This paper uses comparative historical research methods (Wilde 2020; Clemens 2005; Mahoney and Rueschemeyer 2003) to analyze two underexamined sources of data which span nearly 2 decades of American history.

### 2.1 Time Period, 1918–1935

The American religious field in the early 20th century offers a particularly valuable snapshot of class identity and religious life for several reasons. First, the time period covered in our periodical archive (1918–1935) overlaps with the years that the U.S. Census Bureau administered the *Census of Religious Bodies*. This combination of excellent qualitative and quantitative data presents a unique opportunity to examine groups' expressed class identities in relation to their material resources. Second, this is a period in which a number of social upheavals brought social class to the forefront of the American public consciousness. Among many other movements and events (see Supporting Information S1: Appendix A1), the shock of the Great Depression made considerations of class newly unavoidable. As the Depression wore on, Americans were forced to grapple openly with the role of social class and inequality in a way they rarely have before or since (Stillman 2015). This historical context encouraged groups to pay attention to structures of social class and inequality—and articulate where they stood within those structures.

### 2.2 Religious Periodicals

Our study relies on a database of articles from religious periodicals originally compiled by Wilde for *Birth Control Battles* (2020). The sample includes 31 denominations (see Supporting Information S1: Appendix A2), which had a combined membership of over 49 million people—approximately 90% of Americans who were formal members of religious groups in 1926. Religious periodicals sought to educate members about contemporary issues and relate them to the group's beliefs (Haveman 2015). One hundred years later, we use them to explore how each group thought of themselves in terms of social class.

Of Wilde's initial database of over 10,000 articles, approximately 4500 were from our time period. Of these, 1673 contained keywords that we deemed relevant to social class. (The full list of keywords used for this search can be found in the first column of Supporting Information S1: Table A3.) We examined the titles and summaries of these articles, and ultimately close‐read and coded the 893 articles that had the strongest, clearest indications of social class. We also examined 1200 additional articles that had already been transcribed and coded for other projects using this dataset. We undertook an iterative coding process that resulted in the codes listed in the second column of Supporting Information S1: Appendix Table A3. The 84 articles quoted in this paper are the most evocative of the overall pattern of class identity for each group.

On average, we close‐read and coded 30 class‐related articles per denomination, though denominations varied widely in how often they discussed issues related to social class, from a maximum of 308 articles from our two Roman Catholic periodicals, to a minimum of 7 from the Methodist Episcopal Church, South. While the amount of ink spent on topics related to class is relevant to our analysis, we are reluctant to put too much emphasis on this factor, for a number of reasons: First, the length of articles varied significantly from periodical to periodical, with some publications, such as the *National Baptist Union‐Review* and the *Golden Age* publishing mostly items consisting of only a few paragraphs, and others, like the *Methodist Episcopal Quarterly Review* publishing 20‐page essays. Second, two of our periodicals were published quarterly rather than monthly—the *Methodist Episcopal Quarterly Review* and the *A.M.E. Zion Quarterly Review*. Finally, some periodicals were unavailable for some of the years of our analysis, including the Conservative Jewish *S.A.J. Review* and Reform Jewish *Union Tidings,* both of which folded during our study period. Thus, we focus on the character of the class identities expressed in these periodicals, and not on a quantitative assessment of how frequently or powerfully they expressed those identities.

### 2.3 Coding Class Identity

Much debate has occurred over which terms are best for describing the most privileged segments of American society, and whether one needs to own the means of production to fall into the highest categories in the hierarchy (c.f. Lareau and Conley 2008; Wright 2005; Armstrong and Hamilton 2013). Rather than taking sides in this debate, we sought to identify terms that captured denominations' own subjective understanding of their class position. Explicit uses of the word “class” were quite rare among the two more privileged groups in our sample, so we have labeled them the “Professionals,” and the “Property Holders,” which were concepts these groups used to describe themselves. Similarly, we borrowed the emic term “Laborers” to describe the class identity of the less privileged group of denominations. We argue that the consistent portrayals of class identity we find within each group are reflective of a tacit agreement (at least on the part of editors) as to what kinds of people their periodical served, *not* that members were all of this social class or necessarily identified with the class identity broadcast by their denominational periodical. Rather, we intend to show that religious organizations had class identities in the early twentieth century, and that these identities reflected the overall material realities of their constituencies at the group level.

### 2.4 The Census of Religious Bodies

To determine whether the class identity categories above aligned with groups' material resources, we drew information from the 1916 and 1926 editions of the *Census of Religious Bodies* (*CRB*), a decennial census of religious organizations conducted by the U.S. Census Bureau (1916, 1926). The *CRB* are remarkable in their thoroughness and representativeness, ^2^ but until now they have been largely untapped by researchers for anything beyond membership data—mostly because they were not digitized until recently (Wilde et al. 2025).

## 3 Findings

### 3.1 Variations in Wealth Among America's Religious Groups Circa 1926

There was substantial wealth inequality within the American religious field a century ago. Table 1 demonstrates that, on average, the Professionals had nearly twice as much wealth per capita as the Property Holders and more than three times that of the Laborers. Table 2 indicates that the Professionals were also disproportionately urban, with 35% of their members in urban congregations at a time when the U.S. population was only 31% urban (U.S. Census Bureau 1913, 64, *calculated using table 40*). In contrast, the Property Holders were decidedly rural, with 49% of their members worshiping in strictly rural areas. While the rural Property Holders were only half as wealthy as the urban Professionals, they were nevertheless nearly twice as wealthy as the Laborer denominations with $103 in per capita wealth as compared to only $60.

**TABLE 1:** Variations in wealth among American religious groups.

	Members^a^	Total Property Value^b^	Property Value per Member
Professionals			
African Methodist Episcopal Zion Church	456,813	18,515,723	41
American Unitarian Association^c^	60,152	27,713,554	461
Christian Church (General Convention of the Christian Church)	112,795	7,202,193	64
Congregational Churches	881,696	162,212,552	185
Conservative Judaism	—	—	—
Presbyterian Church in the United States of America	1,894,030	338,152,743	179
Protestant Episcopal Church	1,859,086	314,596,738	169
Reform Judaism	—	—	—
Reformed Church in America	153,739	38,436,822	250
Reformed Church in the United States	361,286	44,662,875	124
Society of Friends (Orthodox)	91,326	8,013,407	88
Universalist Church	54,957	15,826,940	288
Jewish Congregations (Aggregate)^d^	4,081,242	97,401,688	24
**Mean** ^e^	**607,674**	**106,313,092**	**201**
Property Holders			
Disciples of Christ	1,377,595	114,850,211	83
Evangelical Synod of North America	314,518	35,789,581	114
Methodist Episcopal Church	4,080,777	406,165,659	100
Methodist Episcopal Church, South	2,487,694	161,986,430	65
Northern Baptist Convention	1,289,966	185,370,576	144
Norwegian Lutheran Church of America	496,707	24,822,215	50
Presbyterian Church in the United States	451,043	67,798,658	150
Southern baptist Convention	3,524,378	173,456,965	49
United Presbyterian Church of North America	171,571	29,714,845	173
**Mean**	**1,577,139**	**133,328,349**	**103**
Laborers			
Assemblies of God, General Council	47,950	3,468,989	72
Church of Jesus Christ of Latter‐day Saints	542,194	15,513,315	29
Churches of Christ	433,714	16,402,158	38
Lutheran Church—Missouri Synod^f^	1,040,275	65,318,781	63
Jehovah's Witnesses^g^	—	—	—
National Baptist Convention U.S.A. Inc.^h^	3,196,623	103,465,759	32
Orthodox Judaism	—	—	—
Roman Catholic Church	18,605,003	837,271,053	45
Seventh‐day Adventist Denomination	110,998	8,477,999	76
United Lutheran Church in America	1,214,340	114,526,248	94
**Mean**	**3,142,068**	**151,568,363**	**60**

*Note:* The bolded values are the means for each category of denomination.

^a^ This figure is drawn from Table 13 of the 1926 *Census of Religious* *Bodies*.

^b^ This figure is drawn from Table 14 of the 1926 *Census of Religious Bodies*. We define “Property Value” here using the value of a denomination's *edifices*, defined as the “estimated value of the church buildings owned and used for worship together with the value of the land on which the buildings stood and the value of the furnishings and other equipment owned by the churches and actually used in connection with church services” (1936 CRB p. 24). This definition goes on the specify that the term excludes investment property, the value of pastors' residences, and the value of school buildings. Therefore, it is a conservative estimate of a denomination's wealth. We debated adding the value of a denomination's parsonages (Table 16) to our property value measure, but found that many denominations were missing this statistic, and including it did not ultimately change the rank of denominations in terms of property value.

^c^ Referred to as “Unitarians” in the 1926 *Census of Religious Bodies*.

^d^ The *Census of Religious Bodie*s does not disaggregate data from Jewish Groups into denominations. In fact, at the time of our study Reform, Conservative, and Orthodox Judaism are better described as movements, rather than formal denominations (Sarna, 2019). However, scholars have argued that Orthodox Jews were the poorest of the Jewish groups in the early 20^th^ century, as they were disproportionately represented among new immigrants (Marcus, 1990; Ellis, 2015; Sarna, 2019).

^e^ The means in our tables omit the statistics from the Black churches, as they deflate the statistics of the “Professional” and “Laborer” groups relative to the “Property Holder” group. We also omit the aggregated Jewish statistics, as they cannot be disaggregated between the three Jewish groups.

^f^ “Evangelical Lutheran Synod of Missouri, Ohio, and Other States” in the 1926 *Census of Religious Bodies*.

^g^ The Jehovah's Witnesses are not included in any of the editions of the *Census of Religious Bodies*. According to the 1936 CRB, “There are certain movements and cults which claim a number of adherents, but are not so organized as to make their presentation as religious bodies advisable. A partial list of these is given below […]” (p. 7) The list includes “Jehovah's Witnesses (Russellites).”

^h^ Referred to as “Negro Baptists” in the 1926 Census of Religious Bodies.

**TABLE 2:** Urbanicity among American religious groups.

	Percent Urban Members^a^	Percent Rural Members^b^
Professionals		
African Methodist Episcopal Zion Church	23	58
American Unitarian Association^c^	57	8
Christian Church (General Convention of the Christian Church)^d^	5	75
Congregational Churches	38	31
Conservative Judaism	—	—
Presbyterian Church in the United States of America	37	29
Protestant Episcopal Church	56	17
Reform Jews	—	—
Reformed Church in America	48	38
Reformed Church in the United States	28	44
Society of Friends (Orthodox)	11	61
Universalist Church	39	25
Jewish Congregations (Aggregate)	95	0
**Mean**	**35**	**36**
Property Holders		
Disciples of Christ	18	45
Evangelical Synod of North America^e^	44	35
Methodist Episcopal Church	23	46
Methodist Episcopal Church, South	10	64
Northern Baptist Convention	40	31
Norwegian Lutheran Church of America^f^	9	75
Presbyterian Church in the United States	23	40
Southern Baptist Convention	8	72
United Presbyterian Church of North America	32	34
**Mean**	**23**	**49**
Laborers		
Assemblies of God, General Council	22	32
Church of Jesus Christ of Latter‐day Saints	19	48
Churches of Christ	5	76
Lutheran Church—Missouri Synod^g^	32	45
Jehovah's Witnesses^h^	—	—
National Baptist Convention U.S.A. Inc.^i^	16	61
Orthodox Judaism	—	—
Roman Catholic Church	57	20
Seventh‐day Adventist Denomination	28	35
United Lutheran Church in America^j^	36	33
**Mean**	**28**	**41**

*Note:* The bolded values are the means for each category of denomination.

^a^ The number of urban members is drawn from Tables 44 and 66 of the 1916 *Census of Religious Bodies*. The 1916 *CRB* defines an “urban” area as town or municipality with over 25,000 members. Using this same definition of urbanicity, the U.S. population was only 31% urban in 1910 (U.S. Census Bureau 1913, 64, *calculated using table 40*). Due to reporting errors, the number of urban members is not always the same between Table 44 (which is reported nationally) and Table 66 (which reports by city). We have defaulted to the higher of the two numbers where both are available. Since this definition of “urban” was only used in the 1916 edition, we have used the 1916 membership numbers (Table 60) to calculate the percentage of urban members. For more on the changing definitions of “urban” and “rural” at the United States' Census Bureau, see Truesdell, 1949.

^b^ The number of rural members is drawn from Table 13 in the *1926 Census of Religious Bodies*. This edition of the *Census of Religious Bodies* defines a “rural” place as a town or municipality with a population of less than 2500 people. To get the percent of rural members, we divided this number by the total number of members in 1926, also from table 13.

^c^ Referred to as “Unitarians” in the 1916 and 1926 *Census of Religious Bodies*.

^d^ Referred to as the “Christian Church (American Christian Convention)” in the 1916 *Census of Religious Bodies*.

^e^ Referred to as the “German Evangelical Synod of North America” in the 1916 *Census of Religious Bodies*.

^f^ The Norwegian Lutheran Church of America was formed between 1916 and 1926 from the merger of Hauge's Norwegian Evangelical Lutheran Synod, the Synod for the Norwegian Evangelical Lutheran Church in America, and the United Norwegian Lutheran Church in America. Our Percent Urban Members statistic is calculated using the sum of the statistics for these three groups in the 1916 *Census of Religious Bodies*.

^g^ Referred to as the “Evangelical Lutheran Synod of Missouri, Ohio, and Other States” in the 1926 *Census of Religious Bodies*. In the 1916 *Census of Religious Bodies,* this group's statistics are not reported separately from those of the Evangelical Lutheran Synodical Conference (of which the Missouri Synod was the dominant member). We have used the statistics of the Evangelical Lutheran Synodical Conference to calculate our Percent Urban Members statistic.

^h^ Although the Jehovah's Witnesses do not appear in the Census of Religious Bodies, existing scholarship suggests that this group was mixed in terms of urbanicity, consisting primarily of small‐scale farmers and industrial workers during the inter‐war period (Penton 2015, 344).

^i^ Referred to as “Negro Baptists” in the 1926 *Census of Religious Bodies*, and the “National Baptist Convention” in the 1916 *Census of Religious Bodies*.

^j^ The United Lutheran Church in America was formed between 1916 and 1926 from the merger of the General Synod of the Evangelical Lutheran Church in the United States of America, the General Council of the Evangelical Lutheran Church in North America, and the United Synod of the Evangelical Lutheran Church in the South. The Percent Urban Members statistic is calculated using the sum of the statistics for these three groups in the 1916 *Census of Religious Bodies*.

These differences in economic and geographic circumstances were clearly reflected in each group's identity. We discuss outliers and marginal cases as they become relevant to our qualitative discussion, below.

### 3.2 The “Professional” Denominations

As members of a communion of religious liberals we represent a definite social class. We are almost wholly bourgeois. We represent the property interests. We are the businessmen of the nation, the professional men, the industrial men and the bankers (Gale 1934, 742).

Twelve denominations in our sample expressed a clear identity as “Professionals.” These include: the African Methodist Episcopal Zion Church, the American Unitarian Association, the Congregational Churches, the Christian Church, Conservative Judaism, the Presbyterian Church in the United States of America, the Protestant Episcopal Church, Reform Judaism, the Reformed Church in America, the Reformed Church in the United States, the Society of Friends (“Quakers”), and the Universalist General Convention.^3^ The Professional denominations saw their people as economically secure urbanites, who imagined their children going to college. They also participated in organized philanthropy, and identified with “industrialists” rather than laborers.

Take, for example, the Unitarians, who described their members as “businessmen,” “professional men” “industrial men,” and “bankers” (Gale 1934, 742), in just one of the astounding 93 articles they published on class‐related topics. The Protestant Episcopal Church (which published 66 articles on class‐related topics), took the side of its “professional” and “salaried” members in an article in the *Living Church*:

With the growing urgency of the conflict between labor and capital, there has grown up a belief that they were the only classes in the community, but our author believes that the class of professional and salaried men has its rights, as apart from capital and labor, which it is in the public interest to protect.

(Woodruff 1924, 468)

If male readers were imagined as urban professionals, female readers were imagined as wives in comfortable urban or suburban households. The Quaker periodical, the *Friend* (which published 48 articles on class‐related topics), described how a group of Quaker women conducted a survey of 10,000 Philadelphia‐area housewives to learn about “problems related to household management,” and the “hours, wages, working and living conditions and social relationships of paid workers in these homes” (“Scientific Management in the Home” 1930, 6). Not only did these women have enough free time, education, and resources to conduct such a study, but the premise of the entire undertaking suggests that Quakers were generally members of a social class that hired household workers.

Occasionally, Professional denominations would explicitly label their members' social class, as the Unitarians did in the quote at the start of this section, when they labeled themselves “bourgeois.” However, it was much more common to use euphemisms like “fortunate,” as when a contributor to the Conservative Jewish *S.A.J. Review* (which published 24 articles on class‐related topics) described his fellow Society for the Advancement of Jews members as, “young people particularly fortunate and in excellent economic circumstances” (Berg 1926, 15). Similarly, an article in the Reform Jewish *Yearbook of the Central Conference of American Rabbis* (which published 41 articles on class‐related topics) bemoaned declining birth rates among the “well‐to‐do classes,” and instructed its primarily rabbinical readers to “urge parents possessed of high‐grade physical, mental and moral qualities and adequate economic resources”—presumably the members of Reform Jewish congregations—“to beget more children” (Frisch et al. 1926).

While these “Professional” denominations projected a clear understanding of the privileged social class of their members, it was rare for them to acknowledge their position in the class hierarchy in explicit comparison to less fortunate groups. In fact, when “Professional” denominations made comparisons, it was almost always as a point of pride relative to other well‐off groups, as in one *Christian Register* article where Unitarians boasted of the high concentration of “eminent” men among their ranks:

Baptists clergymen fathered 1,105 eminent sons; Methodist clergymen, 495 sons; Presbyterian, 4,235; Episcopal, 5,565; Congregational, 6,000. Data for Unitarians are not given, because the number of our body are small for such a computation; but Visher says—and later it is proved—that Unitarians rank even higher than Congregationalists.

(“Factors in Eminence” 1925, 50)

However, when “Professional” groups did occasionally acknowledge that they had privileges that were inaccessible to the lower classes, they framed those privileges as related to knowledge and culture, not material resources. For example, the Congregational and Christian Church's *Congregationalist and Herald of Gospel Liberty* (which published an astonishing 108 articles on class‐related topics), lamented that tenant farmer families “with twelve children” didn't know about birth control methods: “Why must this knowledge remain a class privilege?” (Thompson 1932, 1037).

As part of their privileged class position, the “Professional” denominations talked about higher education frequently, and implied that a substantial number of their own young members would matriculate. This is especially notable given that only 7% of adults ages 18–24 were enrolled in a 4‐year college in 1930 (Snyder 1993, 65). College graduates were discussed as a point of pride. For example, one of the 32 articles we found on class‐related topics by the Reformed Church in America noted that “a high percentage of our ministry are college graduates. The implications of that are weighty” (“The Point of View: Misled but Benefitted” 1929, 760).

Many articles focused on the spiritual needs of college students, and the concern that they would stray from the church. A contributor to the *Living Church,* wrote that:

Every June we are graduating from our institutions of higher learning thousands of young men and women who will be the leaders in the professional, business, and social worlds, who are absolutely divorced from the Church […] young pagans […] with no theocentric orientation whatsoever.

(Wood 1931, 857)

The Presbyterian Church in the United States of America (which published 44 articles on class‐related topics) affirmed the Presbyterian history of Princeton University and called for its recommitment to religious instruction in two articles in *Presbyterian Magazine* (Dodds 1935, 1; “A Princeton Seminary Appeal” 1935, 18).^4^ This focus on the inner workings of college education in general and elite schools like Princeton in particular shows how these periodicals understood their readers to be the kinds of people who go to elite colleges.

Despite their concerns, Professional denominations were confident in the utility of college education, as when the Reformed Church in the United States (which published 40 articles on class‐related topics) wrote of an “impressive” “gathering of American college students” in the *Reformed Church Messenger,* and asserted that “The future of the country lies more fully perhaps with them than with any other equal body of persons that could be brought together” (Elson 1924, 30–31).

Philanthropy also emerged as an important topic that the Professionals focused on much more than other groups. These articles framed readers as people who could use their resources to help others, not as the unfortunate people in need of this kind of help. For example, even during the depths of the Great Depression, the Reformed Church in America's *Intelligencer‐Leader* still drew distinctions between themselves and the needy in this curiously detached statement: “One of the benefits of the depression has been that we have all had our incomes so reduced that we can sympathize with the needy as we may never have been able to do before” (Meulen 1935, 16).

Notably, Professional groups tended to highlight organized large‐scale philanthropy, as opposed to simple charity. For example, a contributor to the Conservative Jewish periodical, the *S.A.J. Review,* wrote to promote the Junior Federation of Jewish Charities, expressing his wish that “the rising generation […] may participate intelligently in the administration of the Federation's agencies” (Berg 1926, 15). The Protestant Episcopal Church's periodical, the *Living Church,* praised the newly‐founded Philadelphia Foundation for its role in making sure that the funds of “wealthy, generous citizens” would be “properly administered” (“Philadelphia Churchwomen on the Housing Problem” 1919, 608). The *Christian Leader* (which published 43 articles on class‐related topics), highlighted Universalists' history of major philanthropy as a point of pride:

Clara Barton, the founder of the American Red Cross Society, was a Universalist. Horace Greeley, Mary A. Livermore, Peter Cooper, and men and women of similar philanthropic fervor were and are members of this church.

(Rose 1926, 8–9)

This preoccupation with organized philanthropy, as opposed to simple charity, is less pronounced in the Property Holders' periodicals and does not appear in the Laborers' periodicals at all.

The Professional denominations' privileged identity also becomes clear in their discussions of labor conflict, in which they often expressed sympathy for industrial workers, but ultimately identified more with capital and management. Even groups seeking to peacefully resolve conflicts and improve conditions for workers sought to do so through their close relationship with management, not labor, as in the following example from the *Christian Leader*:

[T]he time has come for […] brotherly relations in business establishments. Discipleship to Christ in business must take this form. A new evangelism must come into being, an evangelism which impels men holding positions of influence or possessing great economic power to devote themselves and their resources to human welfare, as a part of the Christian program.

(“Labor Sunday Message, 1929” 1929, 945)

Here, the implication is that Universalists are those “men holding positions of influence” with “great economic power.” Likewise, an article in the Unitarian *Christian Register* argued that “church leaders who, in the main, deal with the master group,” should “bear in mind […] problems of industry” (“Economics and Industry” 1930, 10).

The confluence of economic privilege, position in the labor market, familiarity with education, and charitable impulses can be seen in an especially evocative article in the *Congregationalist*, titled “Facing the Down and Outs”:

Who was I that I might enjoy a beautiful home in the suburbs, with dollars in the bank and a place in society, while these men were homeless, dollarless and with no place to lay their heads except what charity provided? Why was my boy, studying his Latin for tomorrow’s test, at my desk surrounded by books, while this boy over in the corner, who cost his mother as much pain and who came into being at as much cost to creation, was bleary‐eyed and facing a future of crime and remorse?

(Morgan 1929)

The author (a minister with a Doctor of Divinity degree) is a secure professional, whose child is preparing for a classical college education by taking courses emphasizing Latin. He lives a comfortable suburban lifestyle, but remains close enough to the urban context to observe and bemoan urban poverty.

#### 3.2.1 Urban Identity

This brings us to a key aspect of the Professionals' class identity—their familiarity with urban life. In 1925, the *Living Church* asserted that “the Episcopal Church has the reputation of being an urban Church with an urban point of view.” (“The Social Service Conference at Manitou” 1925, 246). Ten years later, the same denomination noted that “The urban characteristic in the membership of the Episcopal Church is observable in *Who's Who* [A directory of prominent Americans], particularly the number born in cities of 250,000 and over” (White 1935, 295).

Professional denominations generally expressed familiarity with urban social, economic, and political issues. For example, the *Reformed Church Messenger* wrote favorably about publicly funded municipal housing projects for laborers, arguing that privately funded housing projects weren't feasible: “[E]very economist knows that the adequate housing of the poor is hardly ever a profitable investment […]” (MacCallum 1931). The article continued, “Surely it is part of the Church's task to inspire such far‐visioned leadership in industry and commerce as to give every man in the community the opportunity to work” (MacCallum 1931). This displays not only their familiarity with urban social issues, but also their perspective as investors in the business aspects of city life.

Many Professional denominations also wrote in detail about happenings in specific urban centers. For example, *Presbyterian Magazine* mentioned activities in New York, Philadelphia, and Washington, (Lutz 1924, 596); the Unitarian periodical mentioned events in Boston (“The Future of Temperance” 1919: 598) and Detroit (Davidow 1933); Conservative Jews spoke of the Bronx (Finkelstein 1930, 15); and the Universalists, Detroit (Herring 1926). The Reformed Church in America wrote about their building complex on 22nd Street in New York City (Drukker 1935), but also activities in regional cities like Des Moines (“Christianizing a City” 1925) and the Congregational Christian Churches wrote about their “Pilgrim Faith in Pittsburgh” (1932, 1434).

Others expressed a more suburban identity—close enough to the city to be familiar with its happenings, but safely removed from the worst of its problems, like the above‐cited Congregationalist with his “beautiful home in the suburbs” who nevertheless found himself “Facing the Down and Outs” of the city (Morgan 1929). This situation is especially evident among the Friends, who have an urban‐suburban identity even though only 11% of their people were members of urban congregations (see Table 2): One contributor to *The Friend* describes a day spent “idling” in Herald Square (without feeling the need to specify that Herald Square is in New York City) contemplating the “polyglot group” of immigrants gathered there, and lamenting their exclusion from American social life: “How many generations will it take to admit him to spiritual membership?—to give him the real spiritual freedom of the city?” (Norment 1924a). *The Friend* also reported happenings in suburbs such as Swarthmore, PA (Norment 1924b).

So far, we have argued that the Professional class identity emerged from a combination of wealth and urbanicity. However, there are some outliers that seemingly do not fit this pattern, but that nevertheless support our argument. Most notably, the African Methodist Episcopal Zion (A.M.E.Z.) Church expressed a Professional identity, despite being seemingly too poor and too rural to fit the pattern (See Tables 1 and 2). The A.M.E.Z. Church was a Black denomination and was therefore disadvantaged relative even to the poorest White denominations in our study. However, the contents of the *A.M.E.Z. Quarterly Review* (which published 26 articles on class‐related topics) reveal that this group considered their people to be among the most privileged Black people in the country. They expressed familiarity with higher education and the expectation that their members would pursue it, as when one article asked how the Church should face “the large number of young people who are now attending colleges” (Grimke 1930, 5). Another article showered praise on “great Negro schools” like the Tuskegee Institute while reminding readers that the chance to attend such institutions was still a “special privilege” (“Notes and Comments” 1930, 42–43).

Calls to uplift the less fortunate members of the race were also an important topic in the *A.M.E.Z. Quarterly Review*. Discussing the high rate of illiteracy in Washington, D.C., one article noted that “[t]he Negro must not forget we contribute our full quota to that unfortunate figure,” before declaring that “[n]o stone should be left unturned” in efforts “to see that we have a higher educational standard […]throughout the rank and file of our group” (Medford 1930, 6). Here, the implication is that readers constitute a stratum above the “rank and file” of most Black Americans. The A.M.E.Z. was indeed wealthier than the other Black denomination in the sample, the National Baptist Convention, with 20% more per capita wealth. Of course, our sample only has these two Black denominations, so the power of this comparison is limited.

Additionally, despite being 58% rural, the African Methodist Episcopal Church had an urban identity—they praised the “obscure workers in the slums of our cities […] spending themselves with unabated zeal and unrecorded bravery” (Carrington 1924), and highlighted happenings in Harlem (Mason 1929, 4) and the District of Columbia (Medford 1930, 6). This seemingly incongruous urban identity may be partially explained by the fact that the A.M.E.Z. was founded in New York City with the explicit goal of expanding to serve Black Americans in the South (Hood 1895, 56). Though by no means urban in comparison to other Professionals, they were urban in comparison to the National Baptist Convention.

Another special case of Professional class identity concerns the Christian Church, which is especially poor and rural compared to the other Professional denominations. The Christian Church merged with the Congregationalist Church in 1931 after prolonged negotiations, to become the Congregational and Christian Churches. As this merger approached, the *Herald of Gospel Liberty* (which published 35 articles on class‐related topics before 1931), began to adopt the urban identity of the Congregationalists, educating members on urban social issues, such as the horrors of “the receiving ward of a great city hospital,” where “the bruised and maimed and dying are carried in from the shops and the streets […] because factory and mine owners have loved dividends and great profits more than they have loved their fellow men” (“Concerning Infant Damnation,” 1924, 1036). The Christian Church thus represents a rare case of an organization with an identity in transition—still rooted in its rural background, but stretching toward a more urban and upwardly mobile Professional identity.

Taken together, the Professional denominations' identities as privileged urbanites align remarkably well with their material resources, and the outliers have characteristics that suggest they may be exceptions that prove the rule. In the next section, we move on to the Property Holder denominations, which had similarly privileged class identities, but with a much more distinctively rural character.

### 3.3 The “Property Holder” Denominations

Even granting that he shares the property holders' viewpoint more than that of the wage earner, […] there is […] sympathy with human need in terms of economic welfare […] (Taylor 1929, 27).

Nine groups in our sample expressed “Property Holder” identities: the Disciples of Christ; the Evangelical Synod of North America; the Methodist Episcopal Church; the Methodist Episcopal Church, South; the Northern Baptist Convention; the Norwegian Lutheran Church; the Presbyterian Church in the United States; the Southern Baptist Convention; and the United Presbyterian Church of North America. The Property Holders understood their people to be the wealthy farmers and businessmen who were the privileged class of the rural economy.

For example, an article in the Southern Baptist periodical, the *Christian Index* (which published 38 articles on class‐related topics), reported on the wheat market from the perspective of large‐scale farmers and agricultural businessmen:

May wheat is selling at about $1.11 per bushel in Chicago, which is fully 10 cents above our export basis. This shows that our home market is still on a speculative basis, probably sustained by expectations that the import duty will be raised and that before the end of the year all the domestic crop will be required for home consumption, with the exception of durum wheat.

(Paxon 1924, 20)

An article in the United Presbyterian Church of North America's periodical, the *United Presbyterian* (which published 50 articles on class‐related topics), discussed business ethics using a metaphor drawn from agriculture:

The influence of soil, atmosphere, and climate upon plant and animal life is well known […] As plants and animals are affected by their surroundings, so are people […]. Business men understand how hard it is to conduct their business with absolute honesty among competitors who are not troubled by religious scruples.

(“Life Builders” 1932)

The Presbyterian Church in the United States' periodical, the *Presbyterian Survey* (which published 48 articles on class‐related topics), reported on a spiritual retreat gathering “two hundred Business Men's Evangelistic Groups” in Blue Ridge, North Carolina (McCallie 1932, 639–640). In the *Lutheran Church Herald* (which published 67 articles on class‐related topics), the Norwegian Lutheran Church explicitly attributes their economic successes to their agricultural heritage:

When the Norseman came to America they did not settle down in the large cities to make a living as organ grinders and fruit peddlers. They did not stuff the census of the congested districts of eastern cities, but they looked for good, fertile land, discovered the Mississippi valley, and later the Red River valley and selected the best farmlands in the United States. They have been given credit for being among the desirable citizens.

(“When the Norsemen Came …” 1924: 1508)

For the Norwegian Lutherans, their privileged social class was deeply entangled with their Scandinavian ethnicity and their status as early immigrants to the rural Midwest (framed in stark opposition to more recent waves of urban immigrants).

The Property Holder identity could also be entangled with race, as when Southern Property Holder groups imagined their audience of wealthy White farmers as the descendants of recent slave‐owning ancestors. For example, a contributor to the Methodist Episcopal Church, South periodical, the *Methodist Quarterly Review* (which published 7 articles on class‐related topics) claimed authority to write about Black people by declaring, “I speak as a Southern woman, a descendant of generations of slaveholders” (Hammond 1924, 627). Similarly, the *Christian Index* aimed to counteract “recent efforts to misrepresent the attitudes of the better class of Southern Whites” in an article titled, “How the True Southern White Man Regards the True Black Man” (1924). In addition to portraying a privileged plantation upbringing as relatable—the author characterizes a Black man as being “as familiar a figure around the ‘big house’ as the great white oak in the front yard”—the article also indicates that the author considers himself to be part of the “better class of Southern Whites” he is defending.

As with the Professionals, college featured as a frequent topic among the Property Holders. An article in the Disciples of Christ's *World Call* (which published 56 articles on class‐related topics) detailed the goings‐on at the church's denominational colleges, including a new pipe organ at Drake University and contributions to the endowments of their other colleges (“Board of Education and the Work of Our Colleges” 1925, 52). The Methodist Episcopal Church's *Christian Advocate* (which published 85 articles on class‐related topics), wrote of the importance of Christian higher education: “The Christian college, through its various teachers and counselors, is attempting to help the student attain his maximum best” (Emme 1935, 265). Similarly, the Norwegian Lutheran Church's *Lutheran Church Herald* saw Christian higher education as critical to the mission of the denomination:

The ability of the Lutheran Church to accomplish its great mission in this country therefore depends to a very great extent upon its willingness to maintain a sufficient number of colleges to give us an adequate number of properly trained leaders, clergymen and lay‐men.

(Preus 1934, 1113)

Another article in the same periodical praises the Church's colleges:

Let us thank God that we have in our midst high institutions of learning where the Christian faith is held in its integrity. And let us remember that as members of our Church we have been entrusted with responsibility for their continued maintenance.

(Vigness 1924, 934)

The Norwegian Lutheran Church also boasted about the educational superiority of early Norwegian immigrants, including their early efforts to “establish higher institutions of learning to supply pastors for the pioneer settlements” (“When the Norsemen Came …” 1924: 1508).

In an article titled “A Song of Degrees,” the Northern Baptist Convention periodical, the *Baptist* (which published 54 articles on class‐related topics), celebrated “our Baptist share” of recent graduates receiving “the approving kiss of the universities” (Watson 1924, 552). Although Property Holders shared an interest in higher education with the Professionals, an article titled “Geology and the Liberal Arts Colleges” (Patton 1924, 16) in the *United Presbyterian* suggests a way in which the higher education that Property Holders imagined for their families was different: it was more attuned to rural careers in agriculture, agricultural business, and mining.

As with the Professionals, there was a clear expectation among the Property Holder denominations that members would use their resources to be charitable. For example, an address at the Disciples of Christ's 1920 Convention, reprinted in *World Call*, chided members for failing to give more to the church despite their increasing personal wealth:

This is the richest period in all history, money is dangerously plentiful in America and our people are not at all keeping their increase in giving abreast of their increase in wealth.

(Payne 1920, 28)

Even amidst the Great Depression, an article from a Methodist Episcopal Church's *Christian Advocate* argued that the church was failing to consider the needs of the poor because of the relative wealth of its members:

The churches have run away from the areas of desperate need[…]. The masses of the people are simply outside the pale of organized Christianity and largely because the church people have failed to fairly consider their economic welfare.

(Cushman 1935, 598)

Although they discussed charitable giving, the Property Holder groups did not discuss professionalized philanthropy or any charitable organizations outside the church.^5^

Even Property Holders' infrequent discussions of their own members' hardships reveal a privileged class identity. For example, a female member of the Methodist Episcopal Church wrote that after years of “making a home,” the death of her husband forced her into the workforce during the height of the Great Depression, where she discovered that she was competing with “young, good‐looking girls fresh from college”: “[M]y own musty university sheepskin was worth only a wan smile” (“Crumbs” 1935, 508). This article is remarkable because it indicates that the author is a college‐educated woman (a rare class privilege at the time), and that it was primarily her husband's death, and not the economic downturn, that forced her into the working world.

As with the Professional denominations, women were largely imagined as homemakers and employers of domestic laborers. For instance, an article in the *Methodist Quarterly Review* titled “Male and Female” warns against women's increasing involvement in their husbands' business affairs:

Husband and wife are often both engaged in the business world, and the home is turned over to servants. There is a growing tendency to release the mother to enter the business world and hire a woman of inferior ability to look after the children and the home.

(Steadman 1929, 455)

The labor conflicts discussed in the Property Holders' periodicals were much more rural than those discussed by the Professionals. In the *Evangelical Herald* (which published 28 articles on class‐related topics), the Evangelical Synod of North America expressed their outrage at the acquittal of unionized coal miners in Herrin IL,^6^ who had been accused of killing strikebreakers:

[T]he verdict leaves the guilt unplaced and the guilty unscathed. […] The baneful effects of such an ending on similar industrial disputes or disturbances in the future can hardly be overestimated”.

(“The Verdict” 1923)

As with the Professional groups, when labor conflict occurred, the Property Holders were by default more aligned with managers and owners. For example, in the article quoted at the beginning of this section, the Disciples of Christ periodical noted that their ministers were more likely to share “the property holders' viewpoint more than that of the wage earner”:

The problems of how to make ministerial sympathy with the needs of labor vocal and persuasive with a pew so largely set in the assumptions of property rights is a problem that knits the brow of more ministers of the gospel than of any other class who help to mould [sic] public opinion.

(Taylor 1929: 27)

While the Property Holders share the Professionals' difficulty identifying with laborers, only the Property Holders discussed their difficulties in terms of “property rights.”

#### 3.3.1 Rural Identity

The Property Holders were proudly rural. The United Presbyterian Church of North America bragged that their founders had been the “backwoodsmen of Pennsylvania” (Thompson 1924, 25), while the Methodist Episcopal Church, South characterized contemporary readers as “rural‐born white gentlemen” (Hardon 1925, 185). The Norwegian Lutheran Church in America wrote in one article that:

Our Church, the Norwegian Lutheran Church of America, was organized in the country. The vast majority of its members were and are country people […] Even after nearly three generations in America, our Church is still 75 per cent rural and 25 per cent urban.

(Stub 1931, 38–39)

The Disciples of Christ similarly gave statistics regarding the rural nature of their denomination in *World Call*:

Seventy out of one hundred of our churches are rural. We have 600,000 members in rural churches.

(Lewis 1924, 5)

An article in the Methodist Episcopal Church's *Christian Advocate* explained that “many rural and village churches, handicapped by lack of money and of leadership, are yet doing a religious educational work so high in quality as to put to shame many more favored city churches” (Miller 1931, 1161). At the height of the Great Depression, a *Presbyterian Survey* article reported on plans for distributing funds to their “large number of rural churches” (Littlejohn 1935).

Some Property Holder denominations were holding on to their proud rural identity, even as more of their members were becoming urbanites. In article in the *Baptist* titled “Anent^7^ the Problems of the Great Cities,” a Northern Baptist argues,

In a large sense, we are still a rural people in our thinking and living. The local church in the city largely thinks and plans and acts as does the church in the village and the town. We are slow to sense the greater, more acute problems of city life and living.

(Finn 1924, 1128)

This quote reflects the fact that the Northern Baptist Convention was in the process of becoming more urban than rural (see Table 2), much like the nation in general, but that it consciously retained its rural identity. Indeed, the *Baptist* heavily featured news items of interest to rural farmers. For example: “A recent questionnaire reveals a demand from the farmhouse for radio programs having more education and less jazz. The man who works the soil is eager to take advantage of informative broadcasting” (“Folks, Facts and Opinion: The farmer seeks intellectual stimulation” 1929). The Evangelical Synod of North America also retained a rural identity, despite being more urban than rural in 1926, as evidenced by an article in the *Evangelical Herald* asserting that, “[c]ountry life with religious support will build up the whole nation” (“Fifth Quadrennial Meeting of the Federal Council II” 1925: 23).

### 3.4 The “Laborer” Denominations

More and more, smaller men are pushed out by the big fellow. First it was the working man by the middleman. Then it was the middleman by the small capitalist […] the whole wealth of the world is being concentrated in the hands of a few (Beskin 1932, 5).

The Laborers understood their members to belong to a class of economically disadvantaged people. They rarely discussed higher education or philanthropy except dismissively, and sided with labor in labor conflicts. And while these groups did make geographically specific statements, they were more focused on their disadvantaged position than on anything characteristically urban or rural about their members. Ten denominations in our sample presented a predominantly “Laborer” identity: the Assemblies of God; the Church of Jesus Christ of Latter‐day Saints; the Churches of Christ; the Lutheran Church—Missouri Synod; the Jehovah's Witnesses; the National Baptist Convention U.S.A. Inc.; Orthodox Judaism; the Roman Catholic Church; the Seventh‐day Adventist Denomination; and United Lutheran Church in America.

The Laborer denominations recognized that the readers of their periodicals were economically insecure. One reader's letter in the United Lutheran Church in America's periodical, the *Lutheran* (which published 61 articles on class‐related topics), urged editors to offer more practical advice to readers who were struggling during the Great Depression:

Maybe you have already considered publishing a budget plan for people with large families and small salaries. I believe it would be gratefully received if you will remember that our children have as much right to decent, sanitary homes as any child of wealth, as much need for medical attention to insure a fair chance for health, and the right to be helped through at least a high school education.

(“Money and Children” 1934, 22)

Another article in the *Lutheran* explained the periodic “explosion[s] of bitter feeling and violent, destructive action” within congregations by stating that “There are people in the church who are facing economic frustrations in their personal lives, who take it out on the church” (Venable 1935: 3). Even before the Depression, the Roman Catholic Church (which published a whopping 301 articles on class‐related topics), spoke often of the day‐to‐day economic concerns of its members in the *Commonweal*, and called upon the state to intervene:

Even such an item as certified milk—almost indispensable in cities if the child is to live—makes inroads into the family budget which the average wage‐earner cannot ignore. The true function of the state, therefore, is […] to do its best to remedy the practical ills of parenthood.

(“Fear of the Unborn” 1926, 427)

Though they often struggled to feed their children, Laborers were confident that they were better parents than the wealthy, whose use of nannies suggested that they did not actually want to raise children. The *Lutheran Witness* (the periodical of the Lutheran Church—Missouri Synod, which published 42 articles on class‐related topics) wrote that, “If, by accident, a child arrives, it must, most usually, grow up without a mother's love; it is entrusted to the care of strangers. Such are the deplorable conditions in “high” society […]” (Spitz 1923).

Similarly, reflections on money (or lack thereof) often turned into a criticism of wealth in general. In these criticisms, Laborer denominations clearly identified their audience as being among the exploited have‐nots. For example, in an article in *America,* the Roman Catholic Church scorned the hypocritical behavior of businessmen who supported Prohibition.

[T]he […] actions of Prohibitionists are something quite different from their speech. They are a pestiferous, political brood hatched by the hens, peruna,^8^ coco‐cola and like cackling creatures, and supported by capitalists who would wring the last trace of energy from the laborer.

(“The Dry Mr. Anderson” 1919, 447)

An article in the Jehovah's Witnesses' the *Golden Age* (which published 32 articles on class‐related topics) titled “2% own 60% of the Wealth” reports this eponymous statistic from the Industrial Relations Bureau with the ominous commentary: “The thing they did not explain is what happens when the 2% take over the rest […] it couldn't be long now” (1932, 433).

As the only Black Laborer denomination in our study, The National Baptist Convention, U.S.A. Inc. (which published 15 articles on class‐related topics), was critical of both class‐ and race‐based power. An article in the *National Baptist Union Review,* approvingly noted that the attendees at the Southern Baptist Annual Convention in 1927 seemed to be less elite than in previous years, describing a “notable absence of display, self‐seeking, worldly‐mindedness, and merriment too often witnessed at religious conventions” (“Editorial: Southern Baptists,” 1927, 4). Nevertheless, in the same article, the National Baptists criticized White Southern Baptists for sending missionaries to Africa while “ignoring millions of that same people right at their doors. That smacks of hypocrisy, does it not?” (4).

The Church of Jesus Christ of Latter‐Day Saints (which published 35 articles on class‐related topics) framed their members' economic struggles as the result of being persecuted for their faith, and therefore a badge of honor:

When a person actuated by a conviction sacrifices the pleasure of home, forfeits the esteem of his dearest friends, abandons voluntarily the means by which he gets his bread, tears himself up by the roots from the soil that has been sanctified by age‐long memories to try a new life in a strange land—these acts imply character.

(Evans 1924, 136)

In sum, while the Professional and Property Holder Denominations highlighted the comfort and privilege of their members, the Laborer denominations highlighted and, in some cases valorized, deprivation and struggle.

When the Laborer denominations spoke of college education they did so dismissively or with suspicion, in ways that revealed that their members were unlikely to be among the college‐educated. For example, the Seventh Day Adventists (who published 103 class‐related articles, mostly criticisms of science and scientists) published an editorial in *Signs of the Times* which read: “While I thoroughly believe in a university education for both men and women, I believe a knowledge of the Bible without a college course is more valuable than a college course without the Bible” (Johnson 1925, 10). Similarly, the *Pentecostal Evangel*, the periodical for the Assemblies of God (which published 21 articles on class‐related topics) published an article by famed anti‐evolution prosecutor William Jennings Bryan, which differentiated their church's faithful members from “the cultured‐men” at the colleges:

A teacher in Bryn Mawr College in Pennsylvania says he does not believe in a personal God and a personal immortality. When the students come to graduate forty‐five per cent of them tell him that they have discarded the cardinal principles of the Christian faith. The reason they do it is because they have come under the influence of the cultured‐men who are their instructors.

(Bryan 1925, 3)

Likewise, the Churches of Christ (which published 12 articles on class‐related topics), differentiated between college education and other, more valuable forms of knowledge in the *Gospel Advocate*: “I admit that I am not a “scholar” in the sense in which my critics use the term […] But I am a reader, and have been for many years; and I claim to have a few God‐given grains of common sense” (M'Gary 1924, 76). The Church of Jesus Christ of Latter‐day Saints made it clear in the *Improvement Era* that their members did not belong to the “educated aristocracy” for whom education (though not explicitly higher education) has always been available (Harris 1924, 613–614). Another article in the same periodical argues that Mormons “may lack the polish that comes from contact with the schools; but this is their misfortune and not a defect of character. The unpolished diamond is still a diamond” (Evans 1924, 136). The Jehovah's Witnesses were less diplomatic: a contributor to the *Golden Age* wrote sardonically of a college professor studying evolution through fossilized sea scorpions: “the sea scorpion of so long ago had about as much sense as a college professor has now” (“Wise Professor Patten” 1932, 437).

While “Professional” denominations attempted to address the problems of labor strife through their influence on capitalists and factory owners, “Laborer” denominations approached these topics from the perspective of the worker. An article in the United Lutheran Church in America's periodical, the *Lutheran,* praised the tangible ways that Prohibition improved the daily lives of labor organizers and factory laborers: “Monday at industrial plants is less blue and headachy. In the frequent and very intense industrial struggles there has been a notable absence of drink‐inflamed mobs” (“The Eighteenth Amendment—Prohibition” 1920: 126). When labor strife erupted, the Laborer denominations took the side of the workers, as when an article in the *Golden Age* condemned the Indianapolis police for shutting down a dance meant to raise funds for seventeen workers fired for their unionization activities:

Persons familiar with the facts are wondering at just what point of time Indianapolis made its escape from the land of the free and the home of the brave, and disengaged itself from the provisions of the Constitution regarding freedom of assemblage.

(“Liberty at Indianapolis” 1930)

An article in the *Lutheran* urged readers to identify with the laborers in these ongoing industrial struggles, even if they themselves were not working in factories: “Before we take snap judgment concerning the demands of labor let us take a look at the toilers down through the ages. Our history has been short. We have all been laborers in this country” (Cooper 1934, 10). This slightly more detached take on labor conflict might be attributable to the fact the United Lutheran Church in America was one of the wealthier Laborer denominations by 1926 (see Table 1).

An article in the Roman Catholic periodical *America* more forcefully identified with the “laboring population” in non‐unionized factories:

Ours is an intensely industrial center. The large laboring population is entirely dependent upon the factories for subsistence. […] It is generally conceded that our wage scale is below standard because the employers are thoroughly organized and will not allow a union of any kind to gain a foothold in their factories.

(Howard 1930, 553)

The article goes on to point out the hypocrisy of capitalists' philanthropy: “Unwilling to pay a living wage, these same employers are willing and generous contributors to the Welfare Association.”

As suggested above, the Laborers were skeptical of philanthropy and charity. Occasionally, they bitterly referenced the significant sums of money that wealthier groups could raise from their members. For example, the Assemblies of God contrasted their lack of pension funds with the abundant resources available to several Professional denominations in an article in the *Latter Rain Evangel*:

Last year the pension fund for ministers of the Episcopal Church which was started ten years ago, was increased to $17,000,000. The Presbyterian Church has a pension fund of $15,000,000. Ministers, missionaries and those engaged in educational work who have served the Church for thirty‐five years are entitled to a pension when they reach sixty‐five without regard to retirement. While we do not covet the riches of others, we have often wished that there were some provisions in Pentecost for our beloved missionaries and ministers in old age, so that they might be relieved of the anxiety of making a living when unable to do so.

(“Here a Little and There a Little” 1927, 17)

Similarly, an article in the *Lutheran* implored members of the United Lutheran Church in America to give to the church, citing the much larger fundraising of several wealthier Professional and Property Holder groups:

When it is remembered that the Episcopalians have over $20,000,000 in their treasury for this purpose; that the Presbyterians have $7,000,000, and are in the midst of a campaign for $15,000,000 more; that the Congregationalists, who have the least of any of the large churches, have $9,000,000 […] the amount set by the Richmond Convention as our goal seems most conservative and almost small.

(“Keep Together in the Pension Plan” 1927, 7)

The Laborers did promote helping the less fortunate, but their primary mechanism was interpersonal, not financial, as this quote from *The Jewish Forum* (which published 18 articles on class‐related topics) shows in its discussion of one Orthodox Jewish Woman's work helping refugees in Baltimore:

With the passing of the May Laws in Russia […]thousands of Russian Jews migrated to America […] they found in Henrietta Szold a kinship which was unfathomable. She seemed to understand their problems and devised practical means whereby they might more readily adapt themselves to their new environment. She enlisted her friends into service, and began evening classes for adults. She taught the bewildered immigrant the language and customs of America. No doubt, she was the first woman to undertake on an organized scale what we today call ‘Americanization work.’

(Kaplitt 1935)

The article goes on to describe how Szold's legacy is being continued by women volunteering as mentors in the Jewish Big Sisters program. These “practical means” of aiding the less fortunate stand in sharp contrast with the campaigns for large monetary gifts undertaken by the Professionals and the Property Holders.

While the Laborer denominations had a clear identity as people without privileges and with more than their fair share of deprivations, this does not mean they embraced “class consciousness” in the Marxist sense. In an article titled “Religious Journals and Class Consciousness” the United Lutheran Church in America periodical, the *Lutheran*, explained that publications like theirs are often called upon to “offset ‘class consciousness,’” but that they utterly rejected the premise of these requests: “we have no caste, at least none that corresponds to politics and industrial problems” (“Religious Journals and the Class Consciousness” 1920). It is worth noting that we found no such debates over the class consciousness of the more well‐off groups in our sample.

## 4 Discussion

In the early 20th Century, religious organizations articulated clear and differentiated class identities: “Professional” denominations identified as privileged, urban, and closely aligned with capital and higher education; “Property Holder” groups expressed a distinctly rural privilege tied to landownership and agricultural business; and “Laborer” denominations highlighted economic deprivation, skepticism toward higher education and philanthropy, and solidarity with labor. These patterns largely aligned with denominations' material conditions as recorded in the *Census of Religious Bodies*.

Although focused on the class identities expressed by American religious groups nearly a century ago, our findings have relevance to many contemporary findings about class identity. In a recent article, Reuning and McElwee (2022) argue that researchers must consider precarity as a key component of working‐class identity. We agree: our findings support the idea that precarity was among the most important if not the most important factor in the class identities of the Laborers, overriding any geographically‐specific identities such as those that divided the Professionals and Property Holders.

Interestingly, geographically specific operationalizations of social class played an important role in the work of an earlier generation of sociologists of religion. In *Philadelphia Gentlemen,* E. Digby Baltzell wrote:

The business and professional community […] has been dominated by Protestantism, which, in turn, is subtly stratified along denominational lines. On the whole, while the educated elite tend to worship in Presbyterian or Episcopalian churches, the Lutheran, Methodist, and Baptist congregations appeal to the urban middle classes and rural farmers. […]Finally, the Protestants within the laboring classes, disenchanted with the middle‐class smugness which characterizes the major denominations, often prefer the more radical sects‐‐the Jehovah’s Witnesses, for example, or the Four Square Gospel Churches, or the Adventists.

(Baltzell [1958] 1992, 224–225)

Here, Baltzell correctly recognizes not only the denominational divisions between the “business and professional community” and the “laboring classes” but also the role that the geographically‐specific professions—the “educated elites” versus “rural farmers”—play in further dividing the “business and professional” category. These findings are also echoed in Arthur Vidich's *Small Town in Mass Society*, an ethnography of a small town in upstate New York. Describing the membership of the churches in the town, he wrote:

[T]here are a few points at which class and church meet. The membership of the Baptist church consists largely of members of the marginal middle class […]. Although all churches include a few prosperous farmers, the majority of these farmers are Methodists. […] The Congregational church commands the greatest percentage of the professional class, but does not monopolize this group. There are no professionals in the Baptist church.

(Vidich and Bensman [1958] 1968, 231)

In a footnote, he adds,

The Seventh Day Adventists are situated on the fringe of the community and appeal to a small regional membership [….] The Catholic Church, also located on the geographical fringe, was built by, and serves about 20–30 local Polish families.

(227)

Like Baltzell, Vidich's work displays a rough three‐part division of religious organizations, between the more urban professional denominations (Congregationalists), the farmers (Methodists and Northern Baptists), and the working class (Adventists and Catholics). Even though the data collected for these works post‐date ours by 20–40 years, they are remarkably consistent with our findings…with one glaring exception.

Both of these 1950s sociologists used a very familiar label for the class status of our Property Holder denominations that did not appear *at all* in our early 20th century periodicals: “middle class.” The modern use of middle class (as in “upper‐middle class” or “lower‐middle class”) can be dated back to academic circles during the time of our study, and more specifically to Dr. T. H. C. Stevenson's social classification system developed for the British Registrar‐General in 1913 (Szreter 1984). However, it isn't clear whether this system reflected people's self‐conceptions, since population‐level surveys didn't begin to ask these sorts of questions until after World War II, by which time 43% of American White men identified as middle class (Centers 1949). Given the ubiquity of the middle‐class self‐identification just 20 years after the end of our study period, we are left wondering why the term did not appear in religious discourse. Many historians argue that widespread middle‐class identification among Americans was largely a product of the post‐World War II economic boom (Bledstein and Johnston 2001; Mills and Russell [1951] 2002), so it could be that the term was simply not yet in widespread use.

There remain many other unanswered questions about the class identities of religious groups. The most obvious is the question of whether our findings apply to the present‐day American religious field. While the resource disparities between American religious groups identified in this paper are remarkably consistent with present‐day findings (Wilde et al. 2018; Keister 2011; Davidson and Pyle 2011), it is unclear whether this corresponds with persistent differences in *class identity*. Class identification is historically contingent (Katznelson and Zolberg 1987; Andersen and Curtis 2012), so it is possible that the close relationship between religious groups' class identities and their material resources in the early 20th century has not endured, especially given the restructuring of the American denominational landscape after World War II, as described by Wuthnow (1988). We encourage scholars to study the class identities of contemporary groups, while recognizing that modern norms of public relations would preclude or at least soften some of the statements we found in our periodicals.

Furthermore, we acknowledge that there are many relevant aspects of class identity that we cannot examine using the data we have. While gender is known to play a role in class identity (Abbott 1987; Davis and Robinson 1988; Baxter 1994; Davis and Robinson 1988; Surridge 2007), the denominations and periodicals in our sample were dominated by men. It is possible that the more female‐dominated organs of religious denominations (such as women's mission boards or charitable ministries) would have expressed class identity differently. Additionally, with only two Black denominations in our sample, we are unable to fully assess the ways in which class identification likely varied by the racial identity of a denomination, though we were able to show that there were variations in class identity within the field of Black churches. Finally, this paper raises the idea that class identity also intersects with immigration: The Lutheran descendants of early Norwegian immigrants held a Property Holder class identity, while the more recent Roman Catholic and Orthodox Jewish immigrants were Laborers. Immigration and ethnicity (especially as they relate to eugenics, c.f. Wilde 2020) were likely salient for other groups' class identities as well, but we did not have space to examine this systematically. While there are many additional axes of inequality to explore, our findings have nevertheless contributed to complex religion theory by demonstrating that class identity is a salient concept for religious groups, related to their geography and material resources.

These findings challenge the tendency of social movement and labor scholars to treat class identity as an individual‐level phenomenon, as well as the tendency of management scholars to downplay the role of social class in group‐level organizational identity. Recognizing organizations as classed actors could deepen our understanding of political mobilization and social movements, ultimately producing stronger models for how inequalities are reproduced within organizations, between organizations, and in society more generally.

These findings also offer a blueprint for integrating class identity into the study of complex religion. Religious denominations are classed actors in their own right, not just reflections or aggregations of their members. Our findings allow us to re‐emphasize the central tenet of complex religion theory: that looking at religion as an independent causal variable is ahistorical and overly simplistic. The intersection of material resources and geography underpinning the class identities explored in this paper are proof that scholars of religion need to continue measuring the complex inequalities embedded in the American religious field.

## Supporting information


Supporting Information S1

## Data Availability

The data that support the findings of this study are derived from resources available in the public domain, including periodicals listed in Birth Control Battles at https://www.ucpress.edu/book/9780520303218/birth‐control‐bat, and the Censuses of Religious Bodies available through HathiTrust: https://catalog.hathitrust.org/Record/100326902?type%5B%5D=all&lookfor%5B%5D=Religious%20Bodies%201916&ft= and https://catalog.hathitrust.org/Record/001408066?type%5B%5D=all&lookfor%5B%5D=Religious%20Bodies%201926&ft=.

## Endnotes

^1^
As an exception to this trend, see Ellis (2015).

^2^
Stark (1992) showed that the *CRB's* membership numbers can be verified against other datasets collected at the time.

^3^
The African Methodist Episcopal Zion Church had a Professional class identity, despite being a Black denomination and therefore marginalized relative to even the poorest White denominations in our sample. Nevertheless, we find that this identity is consistent with their being the most secure and established of the Black churches (Lincoln and Mamiya 1990), as we discuss below.

^4^
Princeton was officially affiliated with the Presbyterian Church in the United States of America until the beginning of the 20th century (Huttenlocher 2024; Kemeny 1998).

^5^
This is consistent with the observation that “philanthropy” was a largely urban innovation in the early 20th century (cf. Gross 2003; Hall 2006).

^6^
Although they appear to be relatively urban in Table 2, the Evangelical Synod's concentration in the Midwest suggests a different kind of “urban” existence—given that the Census defined “urban” as anything more than 25,000 people, many regional mining hubs would have easily met this population threshold (c.f. Hartley and Katz 1922).

^7^
An old‐fashioned word meaning “about” or “concerning”.

^8^
Peruna was an extremely popular patent medicine in the early 20th century. It happened to contain a lot of alcohol, but would remain legal with the passage of Prohibition (Appel 2008), meaning that its owners stood to profit mightily. Hypocrisy indeed!
