# Supplementary material for: “We Represent a Definite Social Class”: The Class Identities and Resources of American Religious Groups in the Roaring Twenties
Source: Br J Sociol. 2026 Jan 31;77(2):372–91. doi: 10.1111/1468-4446.70063 (PMC12950203; doi:10.1111/1468-4446.70063)
Supplement: Supplementary file 1 — Supporting Information S1 [file BJOS-77-372-s001.docx]

**ONLINE APPENDICES**

“We Represent a Definite Social Class”: The Class Identities and Resources of American Religious Groups in the Roaring Twenties

**Appendix A1. Time Period, 1918-1935**

The late 19^th^ and early 20^th^ centuries saw extreme class differentiation and spawned a host of class-based political movements (Shrock 2004, 4; Rogers-Cooper 2021). Among these was the continuation of the late 19^th^ century populist movement, in which farmers organized to combat the power of railroad barons (Slez 2020, 2; Maier 2014). The early 20^th^ century also saw the rapid urbanization of American life, lending visibility to the problems facing the urban industrial working class (Rabinowitz 2015, Katz Stern and Doucet 1982). This urban working class was increasingly made up of immigrants and racial minorities. The first Great Migration brought one million Black Americans from the rural South to the urban North between 1910 and 1940 (Grant 2020, 3-4). The mass immigration of poor Jews and Catholics to northern cities before the Immigration Act of 1924 intertwined the issues of class, religion, urbanicity, and immigration in the eyes of the public (Joselit 2008). A burgeoning socialist movement made claims on the state based on class (Avrich 1984, 55; Kluever 2023), and workers' rights became a central concern of American politics by the 1930s (Lichtenstein 2013, 4). These political movements explicitly by and for the working class in turn created new forms of class warfare from above, including Social Darwinism and the Eugenics Movement, which held that the wealthy owe their success to a biological advantage and are fundamentally more fit than the poor, and promoted birth control use and forced sterilization among “undesirable” populations (Hofstadter 1944, Pohlová 2022, Wilde 2020, Rosen 2004).

WORKS CITED

Avrich, Paul. 1984. *The Haymarket Tragedy*. Princeton University Press.

Grant, Keneshia. 2020. *The Great Migration and the Democratic Party: Black Voters and the Realignment of American Politics in the 20th Century*. Temple University Press.

Hofstadter, Richard. 2016 [1944]. *Social Darwinism in American Thought, 1860-1915*. University of Pennsylvania Press.

Katz, Michael B., Mark J. Stern, and Michael B. Doucet. 1982. *The Social Organization of Early Industrial Capitalism*. Boston: Harvard University Press.

Kluever, Joshua. 2023. "The Golden Age of Pragmatic Socialism: Wisconsin Socialists at the State Level, 1919–37." *The Journal of the Gilded Age and Progressive Era*, 22(2), 204-223.

Lichtenstein, Nelson. 2013. S*tate of the Union: A Century of American Labor, Revised and Expanded Edition*. Princeton University Press.

Maier, Chris. 2014. "The Farmers’ Fight for Representation: Third-Party Politics in South Dakota, 1889–1918." *Great Plains Quarterly*, 34(2), 143–162.

Pohlová, Hedvika. 2022. “Social Impact of the American Eugenics Movement.” *American & British Studies Annual* 15:148–63. doi: [10.46585/absa.2022.15.2437](https://doi.org/10.46585/absa.2022.15.2437).

Rabinowitz, Alan. 2015. *Urban Economics and Land Use in America: The Transformation of Cities in the 20th Century*. Routledge.

Rogers-Cooper, Justin. 2021. "Class Wars: Race, Class, and Violence in the Long Gilded Age." *Critical Sociology*, 47(4–5), 807–817.

Rosen, Christine. 2004. *Preaching Eugenics: Religious Leaders and the American Eugenics Movement*. Oxford ; New York: Oxford University Press.

Shrock, Joel. 2004. *The Gilded Age*. American Popular Culture through History. Westport, Conn: Greenwood Press.

Slez, Adam. 2020. *The Making of the Populist Movement: State, Market, and Party on the Western Frontier*. Oxford University Press.

Joselit, Jenna Weissman. 2008. *Parade of Faiths: Immigration and American Religion*. Oxford University Press.

Wilde, Melissa J. 2020. *Birth Control Battles: How Race and Class Divided American Religion*. University of California Press.

**Table A2. Periodical Titles**

| **“Professionals”** |  |
| --- | --- |
| African Methodist Episcopal Zion Church | *A.M.E.Z. Quarterly Review* |
| American Unitarian Association^[[1]](#footnote-1)^ | *Christian Register* |
| Christian Church (General Convention) | *Herald of Gospel Liberty^[[2]](#footnote-2)^* |
| Congregational Churches | *Congregationalist^[[3]](#footnote-3)^* |
| Conservative Judaism | S.A.J. Review^[[4]](#footnote-4)^ |
| Presbyterian Church in the United States of America | *Presbyterian Magazine* |
| Protestant Episcopal Church | *Living Church* |
| Reform Judaism | *Yearbook of the Central Conference of American Rabbis; Union Tidings^[[5]](#footnote-5)^* |
| Reformed Church in America | *Christian Intelligencer^[[6]](#footnote-6)^; The Intelligencer-Leader* |
| Reformed Church in the United States | *Reformed Church Messenger* |
| Society of Friends (Orthodox) | *Friend; American Friend; Friends Intelligencer* |
| Universalist Church | *Universalist/Christian Leader* |
|  |  |
| **“Property Holders”** |  |
| Disciples of Christ | *World Call* |
| Evangelical Synod of North America | *Evangelical Herald* |
| Methodist Episcopal Church | *Christian Advocate* |
| Methodist Episcopal Church, South | *Methodist Quarterly Review* |
| Northern Baptist Convention | *Baptist* |
| Norwegian Lutheran Church of America | *Lutheran Church Herald* |
| Presbyterian Church in the United States | *Presbyterian Survey* |
| Southern Baptist Convention | *Christian Index* |
| United Presbyterian Church of North America | *United Presbyterian* |
|  |  |
| **“Working Men”** |  |
| Assemblies of God, General Council | *Pentecostal Evangel; Latter Rain Evangel* |
| Church of Jesus Christ of Latter-day Saints | *Improvement Era* |
| Churches of Christ | *Gospel Advocate* |
| Lutheran Church—Missouri Synod^[[7]](#footnote-7)^ | *Lutheran Witness* |
| Jehovah’s Witnesses | *Golden Age* |
| National Baptist Convention U.S.A., Inc. | *National Baptist Union-Review* |
| Orthodox Judaism | *Jewish Forum* |
| Roman Catholic Church | *America; Commonweal* |
| Seventh-day Adventist Denomination | *Signs of the Times; Watchman Magazine; Liberty* |
| United Lutheran Church in America | *Lutheran* |

**Table A3. Keywords**

| ***From Periodical Archive***  **Eugenics**   - Juvenile delinquency/ Crime - Anglo-Saxon - Superior/Inferior - Racial Stock/Blood(line) - Genetics/Heredity - (Un)desirable - “Race Suicide”/Differential birth rates   **Immigration**  **“Social Gospel”**  **The Depression**   - Capitalism/Socialism - New Deal - Social Security   **Labor**   - Labor Unions/ Movement - Workers/Management   **Higher Education**   - College/University/Trade   **Science**   - Evolution/Scopes Trial/Darwin | ***Emergent Codes***  ***Social Class***   - *Professions/Jobs* - *Salary/wage* - *Comfortable/well-to-do/fortunate/etc.* - *Struggling/ poor/ etc.* - *Philanthropy/charity*   ***Geography***   - *Urban/Rural* - *Village/Town/City* - *Farming/Agriculture/Mining* - *Industry/Factories* |
| --- | --- |

1. Referred to as “Unitarians” in the 1926 *Census of Religious Bodies* [↑](#footnote-ref-1)
2. The *Congregationalist and Herald of Gospel Liberty* after 1930. [↑](#footnote-ref-2)
3. The *Congregationalist and Herald of Gospel Liberty* after 1930. [↑](#footnote-ref-3)
4. Unfortunately, the *S.A.J. Review* was not popularly oriented. By 1929, the publication was defunct. [↑](#footnote-ref-4)
5. Reform Jews’ official publication, the *Yearbook of the Central Conference of American Rabbis,* was not popularly oriented. *Union Tidings*, the “official” publication of the Union of American Hebrew Congregations, was available before 1930 and mostly covered the Reform Judaism movement, rather than national news. [↑](#footnote-ref-5)
6. In 1922, the *Christian Intelligencer* merged with the *Mission Field* and became the *Christian Intelligencer and Mission Field*. In 1930, it returned to *The Christian Intelligencer*. [↑](#footnote-ref-6)
7. “Evangelical Lutheran Synod of Missouri, Ohio, and Other States” in the 1926 *Census of Religious Bodies*. In the 1916 *Census of Religious Bodies*, this group cannot be disaggregated from the Evangelical Lutheran Synodical Conference, of which it was the dominant member. [↑](#footnote-ref-7)
